# The Mutational Spectrum of Pre- and Post-Neoadjuvant Chemotherapy Triple-Negative Breast Cancers

**DOI:** 10.3390/genes15010027

**Published:** 2023-12-23

**Authors:** Adriana Aguilar-Mahecha, Najmeh Alirezaie, Josiane Lafleur, Eric Bareke, Ewa Przybytkowski, Cathy Lan, Luca Cavallone, Myriam Salem, Manuela Pelmus, Olga Aleynikova, Celia Greenwood, Amanda Lovato, Cristiano Ferrario, Jean-François Boileau, Catalin Mihalcioiu, Josée-Anne Roy, Elizabeth Marcus, Federico Discepola, Jacek Majewski, Mark Basik

**Affiliations:** 1 Cancer Genomics and Translational Research Laboratory, Lady Davis Institute, Jewish General Hospital, Montreal, QC H3T 1E2, Canada; 2 Department of Human Genetics, McGill University, Montreal, QC H3A 1A4, Canada; najmeh_al@yahoo.com (N.A.); jacek.majewski@mcgill.ca (J.M.); 3 Department of Pathology, Jewish General Hospital, Montreal, QC H3T 1E2, Canada; 4 Lady Davis Institute, Jewish General Hospital, Montreal, QC H3T 1E2, Canada; celia.greenwood@mcgill.ca (C.G.);; 5 Department of Oncology, Jewish General Hospital, Montreal, QC H3T 1E2, Canada; 6 McGill University Health Center, Montreal, QC H3A 3J1, Canada; 7 Hôpital du Sacré-Cœur de Montréal, Montreal, QC H4J 1C5, Canada; j.anneroy@sympatico.ca; 8 Cook County Hospital, Chicago, IL 60612, USA; 9 Department of Radiology, Jewish General Hospital, Montreal, QC H3T 1E2, Canada

**Keywords:** triple-negative breast cancer, chemoresistance, chemotherapy, genomics, exome sequencing, mutations

## Abstract

The response of triple-negative breast cancer (TNBC) patients to pre-operative (neoadjuvant chemotherapy) is a critical factor of their outcome. To determine the effects of chemotherapy on the tumor genome and to identify mutations associated with chemoresistance and sensitivity, we performed whole exome sequencing on pre/post-chemotherapy tumors and matched lymphocytes from 26 patients. We observed great inter-tumoral heterogeneity with no gene mutated recurrently in more than four tumors besides TP53. Although the degree of response to chemotherapy in residual tumors was associated with more subclonal changes during chemotherapy, there was minimal evolution between pre/post-tumors. Indeed, gene sets enriched for mutations in pre- and post-chemotherapy tumors were very similar and reflected genes involved in the biological process of neurogenesis. Somatically mutated genes present in chemosensitive tumors included COL1A2, PRMD15, APOBEC3B, PALB2 and histone protein encoding genes, while BRCA1, ATR, ARID1A, XRCC3 and genes encoding for tubulin-associated proteins were present in the chemoresistant tumors. We also found that the mutational spectrum of post-chemotherapy tumors was more reflective of matching metastatic tumor biopsies than pre-chemotherapy samples. These findings support a portrait of modest ongoing genomic instability with respect to single-nucleotide variants induced by or selected for by chemotherapy in TNBCs.

## 1. Introduction

Triple-negative breast cancers (TNBCs) are defined as those breast cancers that do not express the estrogen receptor (ER) or the progesterone receptor (PgR) and lack ERBB2 gene amplification [1]. Such tumors have a very poor prognosis even in the case of early stage diagnosis; do not respond to anti-estrogen or anti-HER2 drugs; and are treated mainly with chemotherapeutic regimens, usually using anthracycline- and taxane-based backbones [1,2]. One of the key indicators of poor prognosis is the resistance of TNBCs to chemotherapy treatment. In the case of pre-operative neoadjuvant chemotherapy (NAC) treatment, approximately 40–55% of TNBCs respond completely to powerful chemotherapeutic drugs [3,4]. This percentage increased to 65% with the recent addition of immunotherapy [5]. However, if the TNBC tumors are resistant to neoadjuvant treatment, the prognosis is very poor [6,7]. Although metastatic TNBCs may show some treatment response at first, all of them rapidly become resistant to a wide variety of chemotherapeutic drugs, rendering them difficult to treat [1,8].

The search for predictive biomarkers of chemotherapy resistance to identify novel therapeutic targets is one strategy to overcome drug resistance. Unfortunately, the genomic characterization of primary TNBCs has revealed few clues as to the factors underlying drug resistance. Indeed, primary TNBCs are characterized by frequent TP53 mutations (>70%) and a high degree of genomic instability, but with few recurrent actionable mutations [9]. Moreover, the TP53 gene has not been associated with therapeutic response in large retrospective analyses [10,11], and the best biomarkers of response to any drug are BRCA1/2 germline mutations (about 15%), which indicate responsiveness to PARP inhibitors [12,13].

The Q-CROC-3 clinical project (NCT01276899), a multi-center biopsy-based study, was initiated to provide an in-depth analysis of the molecular factors associated with resistance to standard chemotherapy regimens in TNBCs. Patients with primary TNBC consented to paired biopsies prior to and after completing a standard neoadjuvant chemotherapy treatment. The present report presents on the results of a whole exome sequencing analysis of the paired TNBCs as well as the pre-chemotherapy TNBC samples from the Q-CROC-3 protocol.

## 2. Materials and Methods

### 2.1. Patient Accrual

Five hospital centers (4 in Montreal, QC, and 1 in Chicago, IL) enrolled 60 consecutive patients with operable >2 cm TNBCs under a protocol reviewed and approved by the local ethics committees of each institution (Comité d’éthique de la recherche du CHUM, Cook Country Health’s Institutional Review Board Research Ethics Boards of the McGill University Health Center, Comité d’éthique de la recherche de l’HSCM and Jewish General Hospital Research Ethics Office). All research was performed in accordance with local and international regulations and guidelines, and all patients provided written informed consent to participate in this study. Pre- and post-chemotherapy biopsies as well as surgical residual tumors were collected as part of the study [14]. Blood samples before, during and after treatment were also collected [15].

### 2.2. Sample Processing

Tissue processing and histopathological quality control were performed as previously reported [14]. In summary, all samples collected were evaluated by two breast pathologists who determined the cellularity and the presence of necrosis and stromal contamination. Based on this review, a tumor cell cellularity index or percentage was calculated for each core or surgical sample. Samples were approved for DNA and RNA extraction only if the tumor cellularity was ≥50% [16]. The results of a more thorough tissue quality control evaluation of these samples have been published [10]. Simultaneous extraction of DNA and RNA was performed using the AllPrep DNA/RNA/miRNA Universal Kit (Qiagen, Mississauga, ON, Canada) as previously reported [14]. An average tumor cellularity of 80% was obtained for all samples analyzed after microdissection. Germline DNA from buffy coats was extracted using QIAamp DNA Mini Kit (Qiagen, Mississauga, ON, Canada) following the manufacturer instructions.

### 2.3. Whole Exome Sequencing and Somatic Variant Annotation

DNA libraries were prepared using Agilent’s SureSelect protocol as per manufacturer’s instructions. Two samples (Neo42 and Neo28) of these pairs had very low quantities of DNA extracted, and libraries were therefore generated using the Nextera DNA library protocol (Illumina Inc., San Diego, CA, USA). We sequenced DNA from matched lymphocytes in all but one case, in which we sequenced normal breast tissue (Neo31) to use as a control for germline variants. The sequencing was subsequently performed on Illumina HiSeq2000 platforms with 100 base paired-end reads. High-quality trimmed reads were aligned to the human reference genome (UCSC hg19) using the Burrows–Wheeler Alignment tool (BWA 0.7.12). Insertions/deletions (indels) were re-aligned using the Genome Analysis Tool Kit (GATK). PCR duplicates were marked with Picard. Single-nucleotide variants (SNVs) and indels were called by means of the SAMtools software (v1.2). At the end, variants were annotated with ANNOVAR and custom in-house scripts.

We used the following criteria to call somatic variants: not present in matched normal controls, minimum 10 reads, mapping Quality (MAPQ) of reads >40, as well as criteria based on in-house databases generated in the Majewski laboratory and https://gnomad.broadinstitute.org (v2.1.1 May 2019). This database contains whole exome sequencing data for over 1000 tumor/normal samples. Any variant present in more than 2 non-cancer samples was excluded.

For further analysis, we only included somatic mutations that were nonsynonymous SNVs, stopgains, frameshift insertions and deletions, and non-frameshift insertions and deletions not occurring in repetitive sequences and did not consider variants in UTRs or synonymous single-nucleotide variants (SNVs). Final allele frequencies (AFs) were calculated by dividing the AF by the tumor cellularity percentage for each sample. A deleterious variant was called if one of the following criteria was met: CADD-PHRED score > 10, SIFT > 0.5 or Polyphen2 > 0.5 [17,18].

## 3. Results

### 3.1. Clinical Results

Fifty-six patients with biopsy-confirmed TNBC who underwent neoadjuvant chemotherapy (NAC) according to standard local practice in five different centers in the USA and Canada were included in the study. All patients consented to the collection of pre-chemotherapy and post-chemotherapy tumor biopsy samples (or surgical specimens) for genomic analysis. Because of the small size of the biopsies and the variations in tumor content, only a subset of collected samples met the quality criteria and yielded enough genomic material for molecular analyses (Supplementary Materials, Figure S1) [14]. In all, 26 patients had sufficient DNA for whole exome sequencing on either pre- or post-chemotherapy tumor samples, or both. Of these, 10 were matched pre- and post-chemotherapy pairs. A clinical evaluation of tumor size was performed at baseline, at the midpoint of the treatment and after therapy before surgery. The clinical data for these 26 patients are shown in Supplementary Table S1.

To understand the dynamics of tumor response during NAC in residual tumors, we classified the 10 matched pairs according to the mid/endpoint clinical evaluation. In 3 of the 10 matched pairs (Neo07, Neo30 and Neo50), the tumor had not responded to treatment at any point during treatment, and these patients were labeled “non-responders” according to the RECIST criteria [19] (<30% decrease in tumor size at either mid-treatment or end of treatment). In two of these cases (Neo07 and Neo50), the tumors actually grew while on paclitaxel monotherapy. The other tumors showed variable responses to chemotherapy (>30% decrease in tumor size at any timepoint) and were labeled as “responders”.

We further assessed residual disease by calculating the Residual Cancer Burden ((RCB) on a scale of 0–3) as described by Symmans et al. [20]: RCB-0 (complete pathologic response = pCR), RCB-1 (minimal residual disease), RCB-2 (moderate residual disease) and RCB3 (extensive residual disease). An RCB score of 3 was given by default to the two patients who did not undergo surgery because there was evidence of metastatic disease during treatment (Supplementary Table S1). Since the prognosis of patients with RCB-1 is essentially identical to patients with RCB0 [21,22], we grouped these patients together and labeled them as chemosensitive to separate them from patients with RBC score ≥ 2 who were labeled as chemoresistant.

### 3.2. The SNV Mutational Spectrum of TNBCs

**Figure 1:**
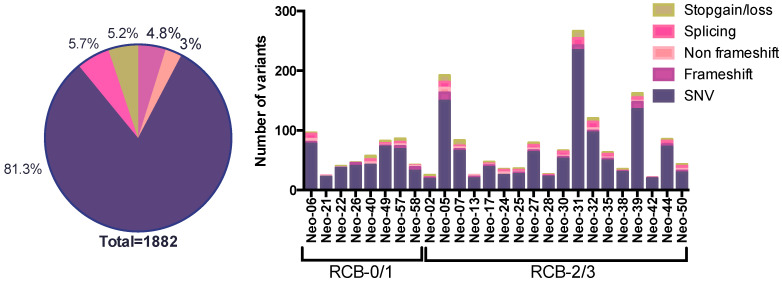
Types and numbers of somatic variants in TNBC tumors. Pie chart with the proportion of each type of mutation identified (left). Bar graph with the number and type of mutation for each patient. The classification according to the response to neoadjuvant chemotherapy is indicated (RCB-0/1 = chemosensitive; RCB-2/3 = chemoresistant).

Whole exome sequencing (WES) was performed on 36 exomes of freshly frozen primary tumors from 26 patients with matched lymphocytes. This includes 23 pre-chemo samples, 10 of which are paired with matched post-treatment tumors, as well as 3 unmatched post-treatment tissue samples. Using our variant calling criteria (see Section 2), we identified a total of 1882 somatic variants in the 36 exomes of primary breast tumors (Figure 1 and Supplementary Materials, Tables S2 and S3). Selected variants (3–5/tumor pair) were validated using digital droplet PCR technology in the same tumor DNA sample as used for sequencing. Every WES variant tested with ddPCR was successfully detected in the tumor DNA and the allele frequencies of 121 variants in the two orthogonal technologies were highly correlated as previously reported (r^2^ = 0.73) [15].

**Figure 2:**
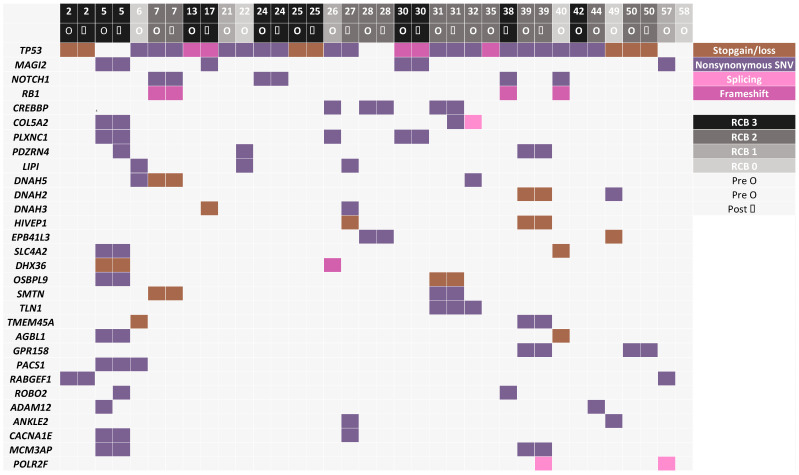
Top 30 mutated genes in tumors from at least 2 patients in pre- and post-chemotherapy tumors (when available). Each column represents one tumor sample, and each color represents different types of alterations. When genes were mutated in only 2 patients, variants with the highest CADD score were selected. RCB score is depicted in grey scale on top of each column.

Using the WES data, we found that three patients were carriers of germline BRCA2 mutations while three patients were carriers of germline BRCA1 mutations. As expected, the most commonly mutated gene was TP53, in 84% of patients (22/26 patients), corresponding to the rate previously reported in TNBCs. The second most commonly mutated genes were MAGI2 and NOTCH1, mutated in tumors from four patients, and seven genes (RB1, CREBBP, COL5A2, PLXNC1, PDZRN4, LIPI and DNAH5) were mutated in tumors from three patients, with no other gene mutated in more than two patients in this tumor set, reflecting the genomic heterogeneity of TNBC tumors (Figure 2). The average number of called somatic mutations was 72/tumor, with a trend toward more mutations in the chemoresistant RCB2/3 group (average 78/tumor) vs. the chemosensitive RCB0/1 group of tumors (average 59/tumor) (*p* = ns) (Figure 1).

We performed three types of analyses: an analysis comparing all pre-treatment samples from “chemoresistant” tumors (RCB2 or 3) to those of “chemosensitive” tumors (RCB0 or 1), an analysis of the post-chemotherapy genomic landscape and an analysis of post vs. pre-treatment matched pairs of tumors to determine changes occurring during treatment.

### 3.3. Mutated Genes Associated with Response to Chemotherapy

**Figure 3:**
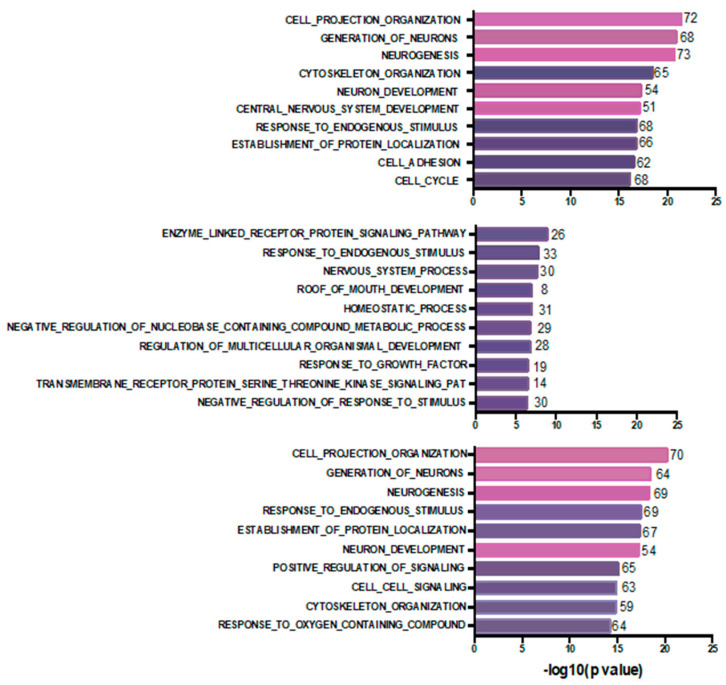
Bar plot for Gene Ontology biological process terms enrichment based on GSEA analysis using the MsigDB [22,23,24]. The top 10 significant terms with FDR < 0.001 are depicted for “chemoresistant” mutated genes in pre-treatment tumor samples from RCB2/3 tumors (**Top**), “chemosensitive” mutated genes in pre-treatment tumor samples from RCB0/1 tumors (**Middle**) and genes mutated in post-chemotherapy residual tumors (**Bottom**). The number of genes overlapping are indicated at the end of each bar. Pink shading highlights terms related to neuron generation processes.

To determine if mutations in the 23 pre-chemotherapy samples could predict chemoresistance, i.e., incomplete response to chemotherapy (“chemoresistance-associated gene variants”), we examined all potentially deleterious variants detected at >0.3 VAF uniquely and only in 15 pre-treatment tumor samples in drug-resistant tumors (RCB2/3), such that none of the mutated genes showed somatic variants in the eight sensitive tumors (RCB0/1). We identified 594 variants in 579 genes (Supplementary Table S4), among these were somatic variants in BRCA1 (in Neo32), XRCC3, ARID1A, ATR and tubulin-associated proteins (TUBE1, TUBA1B, TUBGCP2 and TUBA3E). Reflecting the genomic heterogeneity of TNBCs, only 14 of these genes had variants in more than one tumor and none was mutated in more than two of these tumors (Supplementary Materials, Table S5). To understand the biological functions of the mutated genes, we performed a Gene Ontology enrichment analysis of the top 500 “chemoresistant” mutated genes using MSigDB [22,23,24]. Interestingly, we found that terms associated with cell projection organization, generation of neurons, neurogenesis, neuron development and central nervous development as well as cytoskeletal organization were the top biological processes (BP) associated with these genes (Figure 3).

Analogously, we derived a list of “chemosensitivity-associated gene variants” by combining the list of genes mutated at VAF > 0.3 uniquely in the eight pre-treatment tumors showing complete/near-complete response (RCB0/1) and never mutated in resistant tumors (n = 263 variants in 261 genes) (Supplementary Materials, Table S6). Only two genes were recurrently mutated (COL1A2 and PRMD15). Mutated genes included APOBEC3B, PALB2 and three histone encoding genes (HIST1H2BF, HIST1H2BJ and HIST1H3C). A Gene Ontology analysis of the 261 genes is shown in Figure 3B. Of the top ten biological processes found, only response to endogenous stimulus overlapped with chemoresistant and post-chemotherapy residual tumor gene sets.

### 3.4. Post-Chemotherapy Genomic Landscape

To provide a portrait of the mutational landscape of residual post-chemotherapy samples, we identified all the mutated genes present in the 13 post-chemotherapy tumor samples that had a variant with a minimum VAF of 0.3 (n = 687 variants in 657 genes), a threshold chosen to favor more clonal variants (Supplementary Materials, Table S7). From these 687 SNVs, 612 predicted deleterious missense mutations using liberal thresholds of CADD>10 or SIFT > 0.5 or Polyphen > 0.5, frameshift and stopgain/loss mutations. Besides TP53 and RB1, only 14 of these genes had somatic variants in more than one sample (Supplementary Materials, Table S8). Of these 612 gene variants, we detected 72 variants affecting 66 genes with stopgain/loss variants or frameshift indels, including frameshifts affecting TP53, RB1 and PTEN (Supplementary Materials, Table S9). Interestingly, three genes of the dynein family of microtubule-associated motor proteins (DNAH2, DNAH3 and DNAH5) showed stopgain mutations in different post-chemotherapy residual tumor samples. A Gene Ontology analysis of the top 500 mutated genes revealed that 70% of the identified top 10 biological processes overlapped with those identified in the pre-chemotherapy drug-resistant tumor samples (Figure 3C), with recurrent processes involving neuronal biology. These findings suggest that the mutational spectrum in the chemoresistant tumors can predict the mutational spectrum of post-treatment residual tumors and also suggest that there is little change in the single-nucleotide/small indel-type mutations during treatment.

### 3.5. WES Analysis of Matched Pre/Post-Tumors

**Figure 4:**
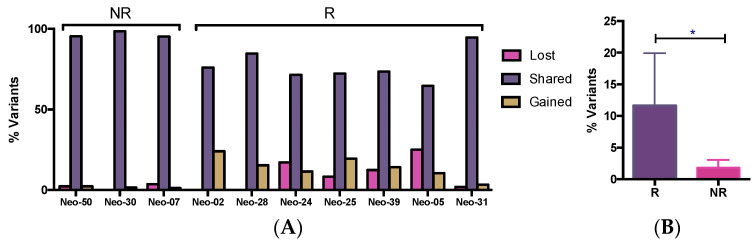
Percentage of variants shared, lost or gained between matched pre- and post-chemotherapy tumors from 10 patients (**A**). Average percentage of variants gained or lost in pre-post chemotherapy matched samples (**B**). NR (non-responder), R (responder). * *p* < 0.05 (unpaired *t*-test).

In the 10 matched pre/post-chemo tumor pairs, a total of 934 somatic variants were observed, with a variable distribution of the number of variants amongst the different tumors. The average number of variants in the pre-chemo samples was the same as that in the matched post-chemo samples (85/tumor). The majority of variants (83%) were present in both the pre- and post-treatment samples (“shared”); only 10% were “lost” (detected only in pre-treatment sample), while 7% were “gained”, i.e., detected only in the post-treatment tumor samples (Supplementary Materials, Table S3). The proportion of variants lost, conserved and gained for each tumor is shown in Figure 4A.

Although limited by the sample size, in an explorative way, we looked at the mutation changes during therapy in the 10 matched tumors, 7 of which were “responders” and 3 of which were previously identified as “non-responders” (Figure 4B). An average of 12% of variants were either lost or gained from pre- to post-chemotherapy samples in the “responder” tumors, while only 2% of variants gained or lost in the “non-responder tumors” (*p* < 0.05). Thus, in the “non-responders”, the “conservation” of variants was consistent with the degree of clinical tumor response, none of which showed any initial response to treatment, including the one tumor that grew while on chemotherapy (Neo07). In the “responders”, although most of the changes (88%) were subclonal (VAF < 0.5), 170 altered genes were enriched (increased VAF by >30%) in the post-chemotherapy sample.

**Figure 5:**
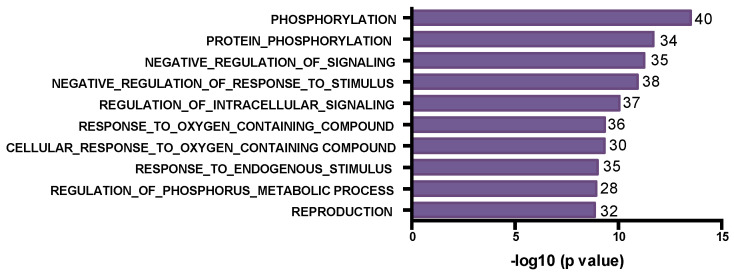
Bar plot for Gene Ontology biological process terms enrichment based on GSEA analysis using the MsigDB [23,24,25]. The top 10 significant terms with FDR < 0.05 are depicted for mutated genes enriched in post-treatment tumor samples. The number of genes in each term is indicated at the end of each bar.

We further looked at the changes in VAF between the 10 matched pairs and identified variants that were enriched for or depleted by treatment in the residual drug-resistant tumors (Supplementary Materials, Table S10). We found 259 genes with increased VAF (>30%), including 3 of the TP53 variants. Interestingly, the most common biological processes associated with these enriched altered genes included mainly phosphorylation, protein modification and signaling-related pathways (Figure 5), suggesting that drug treatment selects for tumor cells containing mutations that mediate drug resistance by altering phosphorylation signaling.

**Figure 6:**
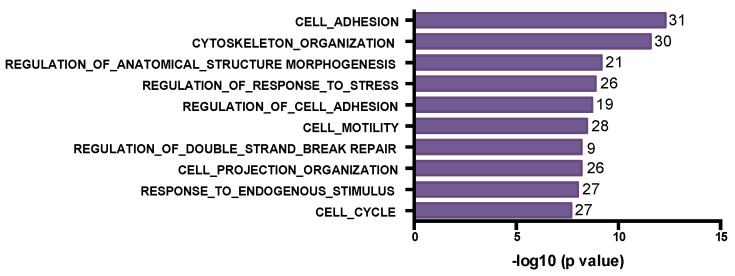
Bar plot for Gene Ontology biological process terms enrichment based on GSEA analysis using the MsigDB [23,24]. The top 10 significant terms with FDR < 0.05 are depicted for mutated genes depleted in post-treatment tumor samples. The number of genes in each term are indicated at the end of each bar.

In contrast, we identified 181 depleted variants, mutated genes with lower VAF (>30% decrease) in the residual tumor. Tumor cells with these mutations are likely sensitive to drug treatment and decrease in proportion in the residual tumor. Cell adhesion, cytoskeleton organization, DNA repair and cell cycle where among the biological processes affected by these mutated genes (Figure 6).

Since the reversion of BRCA germline mutations has been reported to be associated with the acquisition of resistance to chemotherapy in ovarian cancer [25], we also searched for changes in BRCA1/2 mutations and we did not detect any germline mutation reversion.

### 3.6. The Dynamics of Tumor Progression

**Figure 7:**
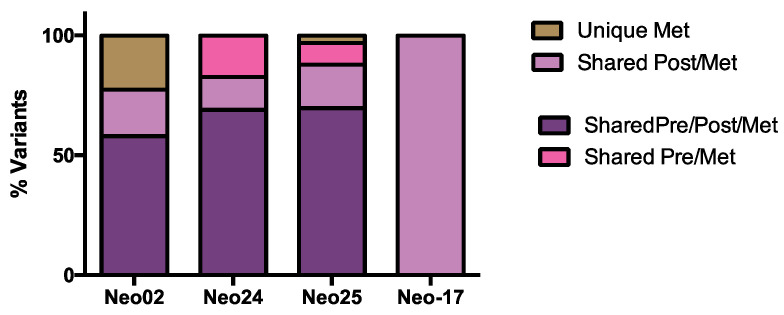
Distribution of variants between primary (pre-chemo and post-chemo) and metastatic samples available from 4 patients. For patient Neo-17, only post-chemotherapy and metastatic samples were available.

In four patients, we obtained frozen tumor samples from metastatic lesions as well as from primary tumors (both pre-chemo and post-chemo tumors except for Neo17 for which only the post-chemo sample was available). These samples were sequenced, and somatic variants detected in the metastatic lesions were compared with the pre-chemotherapy and post-chemotherapy samples (Supplementary Table S11 and Figure 7). We found that the post-chemotherapy samples contained an average of 87% (range 77–100%) of the variants observed in the metastatic samples, compared to 74% (range 58–86%) of the variants in metastases being detected also in the pre-chemotherapy samples. Conversely, on average, 91% of the variants present in the post-chemotherapy tumors were then detected in the metastatic tumor samples, compared with 83% of the variants in the pre-chemotherapy samples. These findings suggest that the post-chemotherapy sample may be a better indicator of the metastatic genotype than the pre-chemotherapy sample. Moreover, these findings, although limited in sample size, suggest that tumor progression is associated with limited appearance of novel single-nucleotide variants in TNBCs.

## 4. Discussion

The treatment of resistant triple-negative breast cancer (TNBC) remains an unsolved problem [8]. Besides PARP and PD-L1 inhibitors, chemotherapy is the mainstay of treatment in TNBCs [2,5]. An excellent clinical setting in which to observe the chemosensitivity of TNBC is the pre-operative or neoadjuvant context. Cortazar et al. [6] confirmed the utility of the response of TNBCs to neoadjuvant chemotherapy as a critical prognostic indicator. In order to advance the care of patients with TNBCs, it is essential to understand the molecular mechanisms of response and resistance to chemotherapy. The objective of the Q-CROC-3 clinical trial was to perform a genomic analysis of pre- and post-chemotherapy tumor samples in the context of neoadjuvant chemotherapy treatment in order to identify mechanisms or markers of resistance and/or sensitivity to chemotherapy. The underlying hypothesis of this experiment was that the treatment of TNBC tumors with chemotherapy would lead to the enrichment and/or selection of genomic alterations that are associated with resistance to the chemotherapy. Here, we present the results of a WES analysis of freshly frozen tumor samples obtained from 26 patients, including data from on 23 pre-chemo samples and 10 matched pre/post pairs.

Although TNBCs are replete with single-nucleotide variants, the appearance of novel SNVs appears to play a minimal role in cytotoxic chemotherapy resistance. WES results showed great inter-tumoral heterogeneity of somatic variants, with the only truly shared mutant gene being TP53. Indeed, each TNBC tumor was essentially unique. We also observe shifts in somatic variants, with a minority of variants being gained or lost during chemotherapy. Clinically “responding” tumors showed significantly greater changes (“losses” or “gains”) of mutated genes than “non-responding” tumors, suggesting that clonal shifts were taking place when tumors were physically changing. However, there were no patterns or recurrently changing mutated genes, and most of these changes were subclonal. Although heterogeneity in tumor sampling may be responsible for some of the changes in VAF in detected variants, the great consistency of variants in the pre- and post-chemotherapy samples in the three matched pairs in which no clinical response was observed suggests otherwise. Indeed, the association of clinical response with an increase in variant gain and losses indicates some genomic evolution during chemotherapy response. Nevertheless, the whole exome sequencing data show that the DNA of TNBCs is remarkably stable during neoadjuvant chemotherapy when the response to chemotherapy is incomplete. These data suggest that there appears to be limited de novo generation of genomically distinct tumor subpopulations during neoadjuvant chemotherapy.

Several other reports reported the results of NGS on post-chemotherapy residual TNBCs. Balko et al. [26] performed targeted sequencing using a 196 gene panel in 74 residual TNBC tumors undergoing neoadjuvant chemotherapy. The authors used formalin-fixed paraffin-embedded tumor material, unlike the frozen biopsies we analyzed; used 20% as a threshold for tumor cellularity; and did not have germline DNA as a control for each tumor DNA sample. They had few pre-chemotherapy samples, and most of the comparisons were with the TCGA database of primary tumors. Potentially actionable gene amplifications were identified in JAK2 (11%) and MCL-1 (54%) in the post-chemo samples. Although their incidence of TP53 mutations was almost identical to ours (89% vs. 84%), we did not identify the same spectrum of mutations in residual tumors. The differences between our results and those of Balko et al. may be due to the lack of germline controls and the use of FFPE samples in the Balko study, or simply to the inherent heterogeneity of TNBCs. Di Cosimo et al. [27] analyzed 19 matched pre- and post-chemotherapy FFPE samples for mutations using a targeted panel of 409 genes. This group also reported few recurrent mutated genes but observed an enrichment in mutations in genes involved in the adaptive immune response in chemoresistant tumors. There were also very few differences in mutations in pre- vs. post-chemotherapy tumor samples. Hancock et al. [28] performed targeted 134-gene sequencing on DNA from FFPE of 18 matched pre- and post-chemotherapy tumor sample pairs and reported no significant gains in deleterious point mutations or indels in these patients, supporting our findings of a relatively stable mutational profile during neoadjuvant chemotherapy. Kim et al. [29] performed a single-cell sequencing analysis in 20 patients with TNBC undergoing neoadjuvant chemotherapy, including 10 with matched pre/post-chemotherapy samples. As in our study, they found few novel mutational changes during chemotherapy and no recurrent “resistance-associated” mutations. More recently, Powles et al. [30] reported a whole exome sequencing analysis of 29 pre-treatment FFPE breast cancer samples including 9 matched pre- and post-chemotherapy pairs, only 3 of which were TNBCs. No germline/normal DNA was used however. As in our study, they also found no recurrent mutations or recurrently mutated genes that were either predictive of response or enriched for in the post-chemotherapy residual tumors. Using the Hallmark pathways for GSEA, they observed that mutations in E2F and G2/M checkpoint-related genes were enriched for in the nine residual post-chemotherapy samples. Finally, Goetz et al. [31] performed whole exome sequencing with germline sequencing in pre-chemotherapy biopsies of 41 TNBCs undergoing neoadjuvant chemotherapy. The most commonly mutated genes were TP53 (76%), PTEN and OTOP1 while variations in the HYDIN and RP genes were enriched for in the chemosensitive tumors. No genomic data from post-chemotherapy samples were reported.

We also performed GSEA analyses of the mutated genes present in chemoresistant tumor biopsies and in the post-chemotherapy residual tumors and found that the biological processes associated with neurogenesis were strongly enriched for. These findings, although unusual, validate our recently reported findings of a novel chemoresistant cellular phenotype in TNBCs [32], in which neuron-like features (e.g., cellular axon-like projections associated with increased MAPT/Tau expression) emerged during bolus doxorubicin treatment of a triple-negative cell line. Interestingly, when performing the GSEA Gene Ontology analysis on differentially expressed pre/post-chemotherapy genes obtained from nine of the Q-CROC tumors included in the present cohort for which RNAseq data were available [32], neuron-related biological processes were significantly enriched for in seven of these nine tumors. Thus, the gene expression data confirm our whole exome sequencing findings. Further studies into this neuronal-like phenotype may shed light on the mechanisms of chemoresistance operating in these TNBC tumors.

Finally, in a small series of matched primary and metastatic tumor samples, we found that the post-chemotherapy sample appears to be a better reflection of the genomic profile of metastatic tumor cells. Nevertheless, the majority of variants detected in our metastatic tumor lesions were already present in both the pre-chemo and post-chemotherapy tumor samples, and most changes were subclonal. This finding appears to suggest that there is limited tumor evolution from the pre-chemotherapy tumor to the post-chemotherapy tumor to the metastatic tumor in TNBCs, consistent with recent reports in which the analysis of metastatic or recurrent TNBCs did not reveal novel driver mutations [33,34]. Although the numbers are small, this may validate the analysis of post-chemotherapy samples (more than pre-chemotherapy samples) for the determination of the metastatic tumor profile. Indeed, the presence of residual tumors after neoadjuvant chemotherapy is a strong indicator of poor prognosis due to the imminent appearance of metastatic disease.

There are several limitations in our study. First, we cannot differentiate the impact of genomic changes on sensitivity to the different individual drugs in the “standard” regimens used in this study. Second, we did not perform whole genome sequencing and thus cannot comment on changes in non-exonic DNA during chemotherapy. Third, we were limited by the small sample size in this biopsy-driven study. Biopsies frequently did not yield enough DNA for whole exome sequencing. The limited number of paired samples in all the studies mentioned above highlights the challenge of collecting residual tumors with enough tumor cellularity for genomic analysis.

## Data Availability

The data supporting the reported results can be found at https://www.ebi.ac.uk/ena accession number PRJEB32259 (accessed on 23 April 2019).

## Supplementary Materials

The following supporting information are available online at https://www.mdpi.com/article/10.3390/genes15010027/s1, Figure S1: Flow chart diagram of Q-CROC-03 study; Table S1: Patient and tumor clinical characteristics; Table S2: Somatic variants detected in the 36 exomes of primary breast tumors; Table S3: Number of variants detected per patient per time point and changes with treatment; Table S4: List of chemoresistance gene variants identified from somatic variants detected uniquely and only in 15 pre-treatment tumor samples in drug-resistant tumors (RCB2/3); Table S5: Chemoresistance genes mutated in more than one tumor; Table S6: List of chemosensitivity gene variants identified from somatic variants detected uniquely and only in 8 pre-treatment tumors showing complete/near-complete response (RCB0/1); Table S7: Mutated genes present in the 13 post-chemotherapy (Post) tumor samples with variant AF (allele frequency) > 0.3; Table S8: Recurrent mutated genes in post-chemotherapy samples; Table S9: Somatic variants affecting 66 genes with stopgain/loss variants or frameshift indels; Table S10. Somatic variants in 10 paired pre- and post-chemotherapy tumors; Table S11: Somatic variants from primary and metastatic matched tumors.
