# Supplementary figures and images for: The Mutational Spectrum of Pre- and Post-Neoadjuvant Chemotherapy Triple-Negative Breast Cancers
Source: Genes (Basel). 2023 Dec 23;15(1):27. doi: 10.3390/genes15010027 (PMC10815241; doi:10.3390/genes15010027)

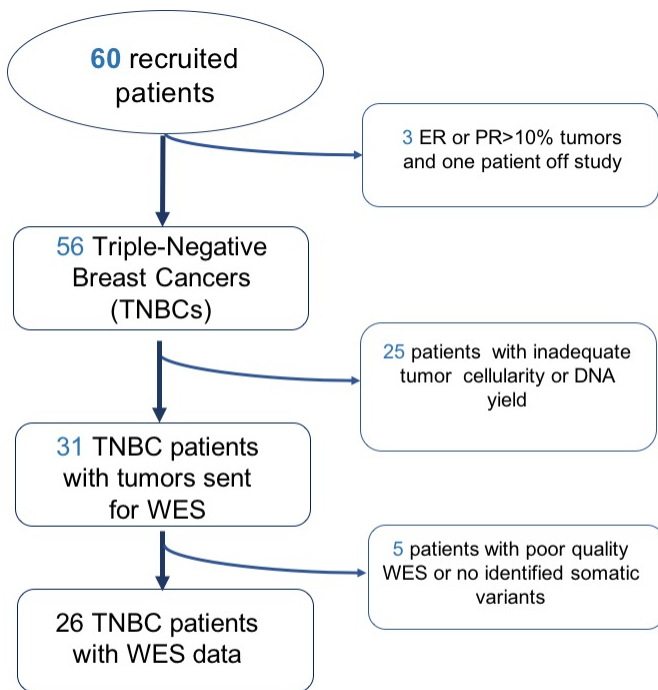

**Supplementary Figure 1. Flow Chart of Q-CROC-03 patient samples included in WES analysis.**

Supplement: Supplementary file 1 [file genes-15-00027-s001.zip › Supplementary Figure S1 GENES.pdf]
